# Biases in facial and vocal emotion recognition in chronic schizophrenia

**DOI:** 10.3389/fpsyg.2014.00900

**Published:** 2014-08-22

**Authors:** Thibaut Dondaine, Gabriel Robert, Julie Péron, Didier Grandjean, Marc Vérin, Dominique Drapier, Bruno Millet

**Affiliations:** ^1^EA 4712 ‘Behavior and Basal Ganglia’ Laboratory, Université de Rennes 1Rennes, France; ^2^Psychiatry Unit, Guillaume Régnier HospitalRennes, France; ^3^‘Neuroscience of Emotion and Affective Dynamics’ Laboratory, Department of Psychology, University of GenevaSwitzerland; ^4^Swiss Center for Affective Sciences, University of GenevaSwitzerland; ^5^Neurology Unit, University Hospital of RennesFrance

**Keywords:** emotional biases, facial emotion recognition, prosody, chronic schizophrenia, vocal emotion recognition

## Abstract

There has been extensive research on impaired emotion recognition in schizophrenia in the facial and vocal modalities. The literature points to biases toward non-relevant emotions for emotional faces but few studies have examined biases in emotional recognition across different modalities (facial and vocal). In order to test emotion recognition biases, we exposed 23 patients with stabilized chronic schizophrenia and 23 healthy controls (HCs) to emotional facial and vocal tasks asking them to rate emotional intensity on visual analog scales. We showed that patients with schizophrenia provided higher intensity ratings on the non-target scales (e.g., surprise scale for fear stimuli) than HCs for the both tasks. Furthermore, with the exception of neutral vocal stimuli, they provided the same intensity ratings on the target scales as the HCs. These findings suggest that patients with chronic schizophrenia have emotional biases when judging emotional stimuli in the visual and vocal modalities. These biases may stem from a basic sensorial deficit, a high-order cognitive dysfunction, or both. The respective roles of prefrontal-subcortical circuitry and the basal ganglia are discussed.

## INTRODUCTION

Chronic schizophrenia is a disabling disease that encompasses both cognitive and emotional disorders (Irani et al., 2011). In recent decades, emotion recognition impairments have received particular attention (Tremeau, 2006), not least because of their impact on social functioning (Kee et al., 2003). A better understanding of emotional impairment in chronic schizophrenia could lead to new therapies (e.g., emotional remediation) and improve social functioning (Penn et al., 2009).

The processing of emotional stimuli in schizophrenia has been investigated in several modalities. Some studies, for instance, have explored the visual modality with faces (see Kohler et al., 2010, for review and meta-analysis) while others have used vocal stimuli, such as emotional prosody (see Hoekert et al., 2007, for review and meta-analysis). These two meta-analyses revealed constant and replicable impairment of the perception of emotional faces and prosody and some clinical features, such as subtype, severity and medication, were found to be related to the impaired perception of emotional faces or prosody.

In addition, few studies have adopted an approach across sensorial modalities, exposing the same patients to both vocal and facial stimuli in two independent tasks. In their review, Edwards et al. (2002) examined seven studies featuring both facial and prosodic emotion recognition tasks. Results revealed a deficit in emotion recognition in both modalities, but highlighted several methodological issues, including clinical and demographic features. In particular, none of these studies examined the results for categorical emotions but used an overall score. Furthermore, the issue of subtype of schizophrenia is important because patients with early schizophrenia showed different performance than patients with chronic schizophrenia. For example, Edwards et al. (2001) identified a specific deficit in the recognition of fear and sadness in both modalities, in patients experiencing their first episode whereas Ramos-Loyo et al. (2012) demonstrated a specific deficit for fear across the two modalities in a group of patients with a paranoid subtype. As for Kucharska-Pietura et al. (2005), when they examined the recognition of emotion across both modalities in patients in either the early or the late stages of schizophrenia, they found that patients in the late stage of schizophrenia performed worse in both modalities and for all emotions than patients in the early stage and healthy controls (HCs). They concluded that emotion recognition impairments increase as the disease progresses. In daily life situations, we use simultaneously visual and vocal cues for recognizing emotion (de Gelder and Vroomen, 2000). Both patients and controls gain from combining modalities (i.e., visual and vocal; Fiszdon and Bell, 2009) but patients with schizophrenia still show poorer recognition abilities compared to controls when performing multimodal recognition tasks (de Gelder et al., 2005; de Jong et al., 2009). In order to attain a further understanding of emotion recognition impairment in schizophrenia, other studies have sought to identify recognition *biases*. An emotional bias is a systematic deviation in the emotional processing. Several studies have examined biases in emotional recognition in facial modality. In patients with chronic schizophrenia, Kohler et al. (2003) described an emotional bias resulting in the over-attribution of disgust and the under-attribution of happiness when labeling neutral faces. Habel et al. (2010) found that, compared with HCs, patients with schizophrenia over-attributed fear or anger to neutral stimuli. Premkumar et al. (2008), meanwhile, described an over-attribution of fear to angry expressions in a mixed population of outpatients with schizophrenia and schizoaffective disorders. In another study, men with chronic schizophrenia overattributed anger to neutral faces, whereas neutral faces were mistaken as sad faces by women with chronic schizophrenia (Weiss et al., 2007). Moreover, it has been suggested that patients with paranoid schizophrenia categorize neutral faces as angry, whereas patients with non-paranoid schizophrenia categorize them as sad (Pinkham et al., 2011). Only a few number of studies focused on bias in emotional recognition using vocal modality. Shea et al. (2007) showed that only patients with schizophrenia with auditory hallucinations are more likely to misattribute emotional prosody to neutral stimuli than patients without auditory hallucinations and HCs. Finally, a recent study investigated biases in emotion recognition in patients with stable schizophrenia in two modalities (i.e., visual or vocal) and multimodal settings (i.e., both visual and vocal; Thaler et al., 2013). In this study, patients exhibited a negative over-attribution during the vocal recognition. For a stimulus considered as neutral, Thaler et al. (2013) showed a negative over-attribution for the visual modality and a positive over-attribution for vocal and multimodal task in a group of patients with chronic schizophrenia. However, in this study, biases were examined toward only neutral stimuli and it is unclear whether emotional biases are observed across modalities for a large panel of emotional categories.

The present study was designed to test emotional biases in chronic schizophrenia in two modalities (facial and vocal) of emotion recognition. To avoid categorization biases, we asked participants to provide intensity ratings on a set of emotional visual analog scales in two facial and vocal emotion tasks taken from Péron et al. (2010, 2011). This new method allowed intensity rating on target scales (i.e., rating intensity on the emotional scale corresponding to the relevant stimuli) and on several non-target scales (i.e., rating intensity on all the other emotional scales). Consistent with previous studies on emotional biases in the visual modality and vocal modality, we hypothesized that patients with chronic schizophrenia would show more emotional bias (by attribution of greater intensity on non-target scales) in the two sensorial modalities.

## MATERIALS AND METHODS

### PARTICIPANTS

Twenty-three (15 men and 8 women) patients with chronic schizophrenia and 23 (12 men and 11 women) HCs participated in this study. All participants were native French speakers.

Patients who tested as clinically stable were recruited from outpatient units at Guillaume Régnier Hospital (Rennes, France). The diagnosis of schizophrenia was established by a clinically trained psychiatrist according to the Mini International Neuropsychiatric Inventory (Sheehan et al., 1998), based on the criteria of the *Diagnostic and Statistical Manual of Mental Disorders, Fourth Edition* (DSM-IV; American Psychiatric Association, 1994). All the patients were taking antipsychotic medication at the time of testing. Six were also taking antidepressants (selective serotonin reuptake inhibitors).

We also recruited 23 HCs. Inclusion criteria for HCs were no current or past mental illness or any psychotropic treatment.

Exclusion criteria for all participants were neurological and systemic illness, head injury with loss of consciousness longer than 15 min, significantly impaired vision or auditory acuity, and a score below 130 on the Mattis Dementia Rating Scale (MDRS; Mattis, 1988).

Patients and HCs were matched on sex, age, education level and handedness (Edinburgh Handedness Inventory; Oldfield, 1971). The clinical and demographic characteristics of the two groups are set out in **Table 1**.

**Table 1:** Clinical and demographic characteristics of patients with schizophrenia and healthy control participants.

	Schizophrenia patients Mean (SD)	Healthy controls Mean (SD)	Stat. val.	*p-*value
Age (years)	33.92 (7.27)	36.51 (7.23)	*t* = 0.46	0.46
Sex (male/female)	15/8	12/11	χ2 = 0.81	0.37
Edinburgh Handedness Quotient	85.87 (33.05)	85 (32.79)	*t* = -0.09	0.93
Education (years)	12.70 (2.01)	13.13 (1.79)	*t* = 0.77	0.44
Duration of illness (years)	12.43 (6.50)			
PANSS (overall score)	66.30 (18.58)			
PANSS (positive subscale)	10.91 (3.54)			
PANSS (negative subscale)	23.52 (9.11)			
PANSS (general psychopathology)	31.86 (8.64)			
SANS score	46.09 (25.37)			
Neuroleptic dosage (CPZ equivalent)	680.39 (461.56)			
Patients on antidepressants (%)	26.09%			

*Stat. val., statistical values; PANSS, Positive and Negative Syndrome Scale; SANS, Scale for the Assessment of Negative Symptoms; CPZ, chlorpromazine; *t*, *t*-test for two independent groups.*

Written informed consent was obtained from each participant and the study was conducted in accordance with the Declaration of Helsinki. The study was approved by the local ethics committee (CPP Ouest II- Angers; no. 2012/16).

### PSYCHOPATHOLOGICAL AND NEUROPSYCHOLOGICAL ASSESSMENT

The current severity of the patients’ psychiatric symptoms was assessed using the Positive and Negative Syndrome Scale (Kay et al., 1987), which is divided into three subscales (positive symptoms, negative symptoms and general psychopathology), the Scale for the Assessment of Negative Symptoms (Andreasen, 1982) and the Calgary Depression Scale for Schizophrenia (Addington et al., 1992).

In order to assess the relationship between cognitive dysfunction and emotion processing, participants underwent a neuropsychological assessment by a trained neuropsychologist (**Table 2**). This assessment took place in single 1-h session prior to the emotional tasks. We used the MDRS to assess overall cognitive functioning, and the Digit Span subtest of the Wechsler Adult Intelligence Scale (WAIS-III; Wechsler, 1997) to examine verbal short-term and working memory. Processing speed and attention were evaluated by the Digit Symbol-Coding subtest of the WAIS-III (Wechsler, 1997). A battery of tests was used to assess executive functions: the categorical and literal fluency test (Cardebat et al., 1990), a Stroop Test (Stroop, 1935), the Trail Making Test (TMT; Reitan, 1958), and Nelson’s modified version of the Wisconsin Card Sorting Test (MCST; Nelson, 1982). The integrity of the early stages of face perception was verified using the short version of the Benton Facial Recognition Test (Benton et al., 1983). To ensure that the participants were free of auditory impairment, they underwent the Montreal-Toulouse auditory agnosia battery (PEGA; Agniel et al., 1992). Results of the PEGA and the Benton Facial Recognition Test showed that none of the participants had any auditory impairment or prosopagnosia.

**Table 2:** Neuropsychological background of patients with schizophrenia and healthy control participants.

		Schizophrenia patients Mean (SD)	Healthy controls Mean (SD)	Stat. val. *t*-test for two independent groups	*p-*value
MDRS (max. 144)		137.74 (6.64)	141.43 (1.75)	2.58	0.01
Digit span	Forward	5.91 (1.28)	5.74 (0.92)	-0.53	0.60
	Backward	4.87 (1.14)	4.61 (1.47)	-0.67	0.50
Verbal fluency	Categorical (2 min)	24.91 (8.02)	31.13 (6.97)	0.89	0.38
	Phonemic (2 min)	19.52 (7.54)	21.39 (6.65)	2.81	0.007
Stroop	Interference	-0.55 (8.56)	3.85 (8.14)	1.79	0.08
TMT	A (seconds)	46.78 (17.24)	31.96 (10.31)	-3.54	<0.001
	B (seconds)	100.65 (53.63)	64.35 (19.30)	-3.05	0.004
	B–A (seconds)	53.87 (43.92)	32.39 (15.36)	-2.21	0.03
MCST	Categories (max. = 6)	5.87 (0.43)	6 (0)	1.45	0.15
	Errors	2.87 (2.75)	2.70 (7.40)	-0.11	0.91
	Perseverative errors	0.87 (1.49)	0.04 (0.21)	-2.64	0.01
Digit Symbol-Coding (WAIS III)		53.62 (14.31)	74.13 (19.52)	4.06	<0.001
PEGA (max. 30)		29.04 (1.15)	29.34 (0.93)	-0.69	0.49
Benton Facial Recognition Test		46.57 (3.26)	45.61 (5.79)	0.99	0.33

*Stat. val., statistical values; MDRS, Mattis Dementia Rating Scale; TMT, Trail Making Test; MCST, Milner Card Sorting Test; WAIS III, Weschler Adult Intelligence Scale; PEGA, Montreal-Toulouse auditory agnosia battery.*

### FACIAL STIMULI

This task featured two different sets of 56 emotional facial expressions produced by eight actors (four male and four female) per set. Six emotions (fear, disgust, anger, sadness, surprise, and happiness) were depicted, alongside neutral faces. For each emotion, there were four male faces and four female ones, making a total of eight stimuli per emotional category. These photographs were taken from Ekman and Friesen’s Pictures of Facial Affect (Ekman and Friesen, 1978) and the Karolinska database (Lundqvist et al., 1998). Mean luminance, apparent contrast and spatial frequency were adjusted according to Delplanque et al. (2007). Each photograph was displayed until the response of participants on all intensity rating scales. Patients and HCs were assessed with two versions of emotional facial recognition task because patients were included in another follow-up study. Eleven patients with schizophrenia and 11 HCs were assessed with Version A (corresponding to the first set of 56 emotional facial expressions). Twelve patients and 12 HCs were assessed with the Version B (corresponding to the second set of stimuli).

### VOCAL STIMULI

The vocal stimuli were taken from the Montreal Affective Voices database developed and validated by Belin et al. (2008). They consisted of non-verbal affect bursts devoid of semantic content (vowel “ah”). We selected two sets of 35 vocal stimuli pronounced by 10 actors (five men and five women). Seven categories were used in this study (anger, disgust, fear, happiness, sadness, and surprise, plus a neutral stimulus). The mean duration of the stimuli was 1084.87 ms (range: 240–4310 ms) and the mean energy of the stimuli was 73.40 dB (range: 47.5–85 dB). Participants were told that they could listen again to each stimulus as many as six times, by clicking on a button on upper right the computer interface. All stimuli were played binaurally via stereo headphones. For the reasons described above, patients and HCs were assessed with two versions of emotional vocal recognition task. Eleven patients with schizophrenia and 11 HCs were assessed with Version A (corresponding to the first set of 35 emotional vocal expressions). Twelve patients and 12 HCs were assessed with the Version B (corresponding to the second set of stimuli).

### EMOTION RECOGNITION PROCEDURE

We administered two emotional tasks: a facial emotion recognition task featuring facial stimuli and a vocal emotion recognition task featuring vocal stimuli. The facial emotion recognition task was always performed before the vocal emotion recognition task. The procedure was the same for both. Participants were seated in a quiet room, in front of a computer. Each condition (faces or voices) was displayed by an Authorware program.

**FIGURE 1:**
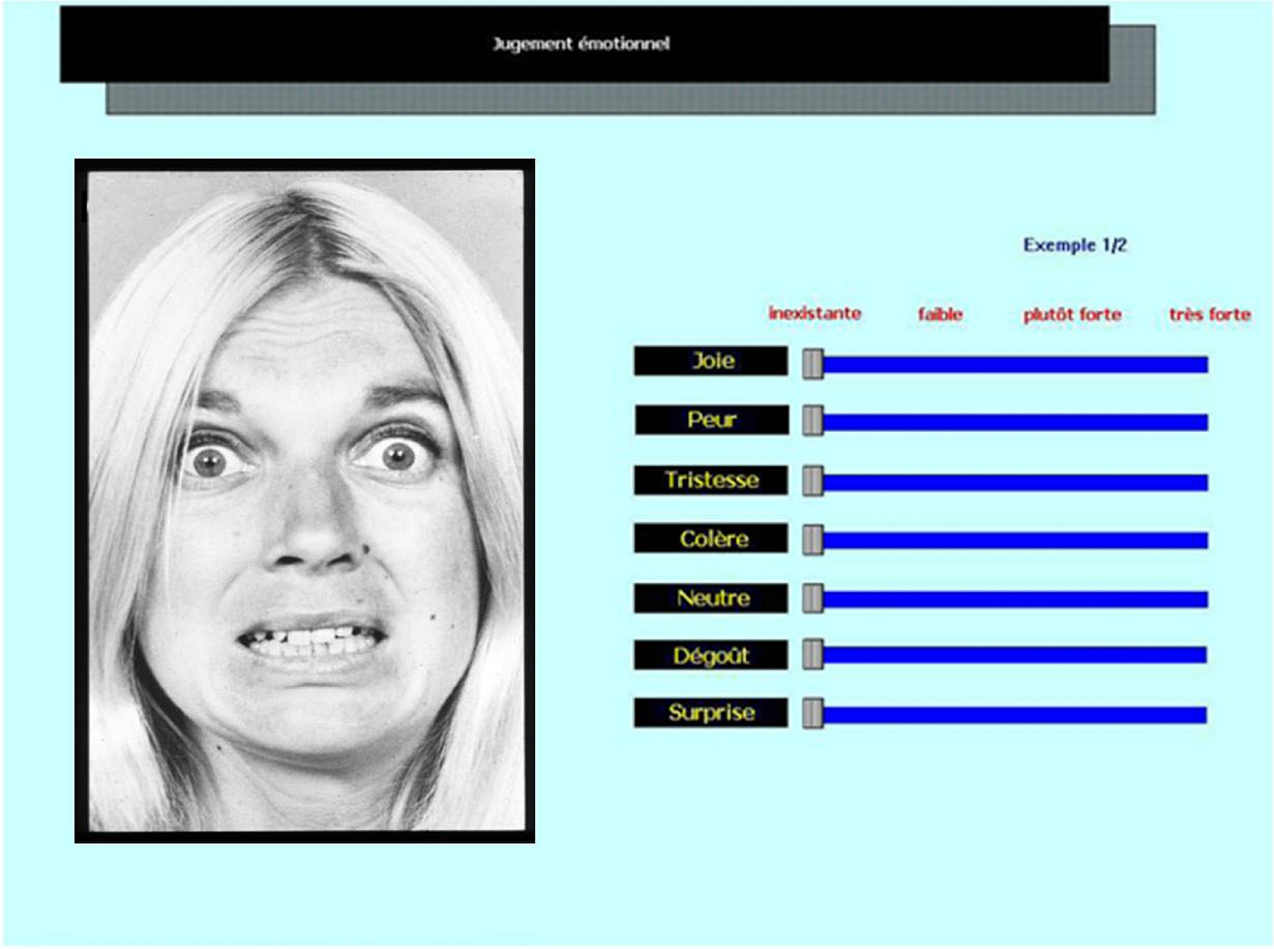
**Computer interface for the facial emotional task**.

At the beginning of each trial, a progress bar appeared on the computer screen. This was followed by the stimulus and participants were asked to rate its emotional content on scales that were simultaneously displayed on the screen. More specifically, participants were instructed to indicate the extent to which the different emotional categories were expressed on visual analog scales ranging from *Not at all* (scoring 0) to *Very much* (scoring 100). Participants therefore rated each stimulus on seven scales (anger, disgust, fear, happiness, sadness, and surprise, plus neutral). When participants completed all assessments of intensity, a button appeared and the next stimulus could be played by clicking on this button. They were given two examples per task, not used in the main task, in order to familiarize themselves with the procedure. An example of the computer interface for the both emotional tasks was provided in **Figure 1**.

The entire protocol was completed in 90 min with a pause between clinical assessments and evaluation of emotion recognition.

### STATISTICAL ANALYSIS

For the two emotion recognition tasks (facial and vocal), we performed two levels of analysis.

#### Categorical judgments

First, we compared the percentages of correct responses for the two tasks. For each trial, we compared the rating of intensity for each scale. A response was deemed to be correct when a participant rated the target scale higher than all the non-target scales. If the intensity were higher for the target scale (e.g., the anger scale for an angry face), we scored “1”; if not we scored “0.” We summed the score for each task and for each emotional category and then, we calculated the percentage of correct responses. We performed a repeated measures ANOVA with group (two levels: patients and HCs) as the between-subjects variable and task (two levels: visual and. vocal) and emotion (seven levels: anger, fear, sadness, disgust surprise, happiness, and neutral) as the within-subjects variable.

#### Intensity ratings

Then, we compared the ratings given by the two groups for each task (facial and vocal) on the scales for each type of emotion and for each individual scale, distinguishing between (1) the target scales, that is, the mean ratings on the scales (e.g., Anger scale) corresponding to the relevant stimuli (e.g., anger stimulus), and (2) the non-target scales, that is, the mean ratings on the scales that did not correspond to the stimuli (e.g., Fear scale for anger stimulus). This second analyses enabled us to take into account target ratings on incorrect responses (e.g., when recognizing an “anger” stimuli, rating 80% on the “anger” scale and “90%” on the surprise scale). In order to pinpoint impaired emotion biases in schizophrenia, we performed contrasts for the non-target ratings for each condition. To this end, we ran repeated-measures ANOVA with group (patients and HCs; two levels) as a between-participants factor, and emotion (seven levels) and scale (seven levels) as within-participants factors. We compared the HC and patient groups on each emotional category and each rating scale.

#### Others

Sociodemographic, psychiatric and neuropsychological data were compared using at-test for two independent groups for two independent groups (patients and HCs).

Versions A and B of the emotional recognition tasks were compared using a *t*-test for two independent groups (version A versus Version B) in the HC group.

Correlations between (1) clinical assessments and daily neuroleptic dose and vocal and facial emotion recognition (2) executive functions performances and the results of the emotion recognition tasks were assessed using Spearman’s rank correlation coefficient for the patients group.

Statistical analyses were performed using Statistica 8.0. The significance threshold was set at *p* = 0.05.

## RESULTS

### NEUROPSYCHOLOGICAL ASSESSMENTS

The patients with schizophrenia scored significantly lower than HCs on the MDRS (*t* = 2.58; *p* = 0.01), categorical fluency (*t* = 2.8; *p* = 0.007), TMT Part A (*t* = -3.54; *p* < 0.001), TMT Part B (*t* = -3.05; *p* = 0.004), TMT B–A (*t* = -2.21; *p* = 0.03), MCST perseverative errors (*t* = -2.64; *p* = 0.01), and the Digit Symbol-Coding subtest (*t* = 4.06; *p* < 0.001), but not on any of the other cognitive tests (see **Table 2**).

### EMOTION RECOGNITION TASKS

#### Analysis of categorical judgments (**Table 3** and **Figure 2**)

**Table 3:** Number of correct responses, expressed as a percentage of total responses (standard errors, SE) for categorical judgments in the facial and vocal emotion recognition tasks of patients with schizophrenia and healthy controls.

		Schizophrenia patients	Healthy controls
		Mean (±SE)	Mean (±SE)
Facial emotion	Anger	68.24 (19.28)	79.34 (20.00)
Recognition task	Disgust	58.93 (27.64)	82.61 (17.57)
	Fear	54.89 (24.35)	52.17 (26.56)
	Happiness	90.22 (14.58)	97.83 (4.84)
	Neutral	70.65 (24.89)	84.24 (17.76)
	Sadness	67.93 (22.87)	72.28 (19.93)
	Surprise	88.04 (20.81)	97.83 (6.14)
	Overall	71.28 (10.93)	80.90 (8.28)
			
Vocal emotion	Anger	41.74 (19.92)	60.87 (17.56)
Recognition task	Disgust	62.61 (30.33)	83.48 (19.68)
	Fear	53.04 (24.58)	73.04 (26.01)
	Happiness	86.09 (24.45)	99.13 (4.17)
	Neutral	74.78 (33.15)	86.96 (21.41)
	Sadness	82.61 (22.81)	92.17 (11.66)
	Surprise	51.30 (30.05)	48.70 (20.74)
	Overall	64.60 (13.91)	77.76 (7.21)

**FIGURE 2:**
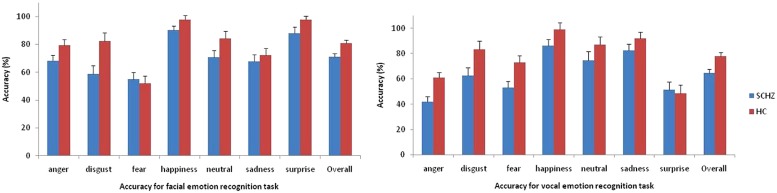
**Accuracy in percentage of correct responses for facial and vocal emotion recognition task. Error bars indicate standard errors of mean**.

Analysis revealed an Emotion × Task interaction [*F*(6,264) = 27.97, *p* < 0.001, partial η^2^ = 0.39]. Contrasts revealed that participants performed better at “anger” [*F*(1,44) = 34.08, *p* < 0.001] and “surprise” [*F*(1,44) = 154.89, *p* < 0.001] stimuli during the facial recognition task. Conversely, participants performed better at “sadness” [*F*(1,44) = 19.95, *p* < 0.001] and “fear” [*F*(1,44) = 5.31, *p* = 0.03] in vocal recognition task.

Analysis revealed a main effects of task [*F*(1,44) = 17.30, *p* < 0.001, partial η^2^ = 0.28] and a main effect of emotion [*F*(6,264) = 25.38, *p* < 0.001, partial η^2^ = 0.37]. A main effect of group was observed [*F*(1,44) = 12.58, *p* < 0.001, partial η^2^ = 0.22]. Patients were less accurate than HC’s.

There was no Group × Emotion interaction [*F*(1,44) = 1.75, *p* = 0.13, partial η^2^ = 0.04], no Group × Task interaction [*F*(1,44) = 1.64, *p* = 0.21, partial η^2^ = 0.04] and no Group × Task × Emotion [*F*(6,264) = 1.93, *p* = 0.10, partial η^2^ = 0.04].

#### Intensity ratings

**FIGURE 3:**
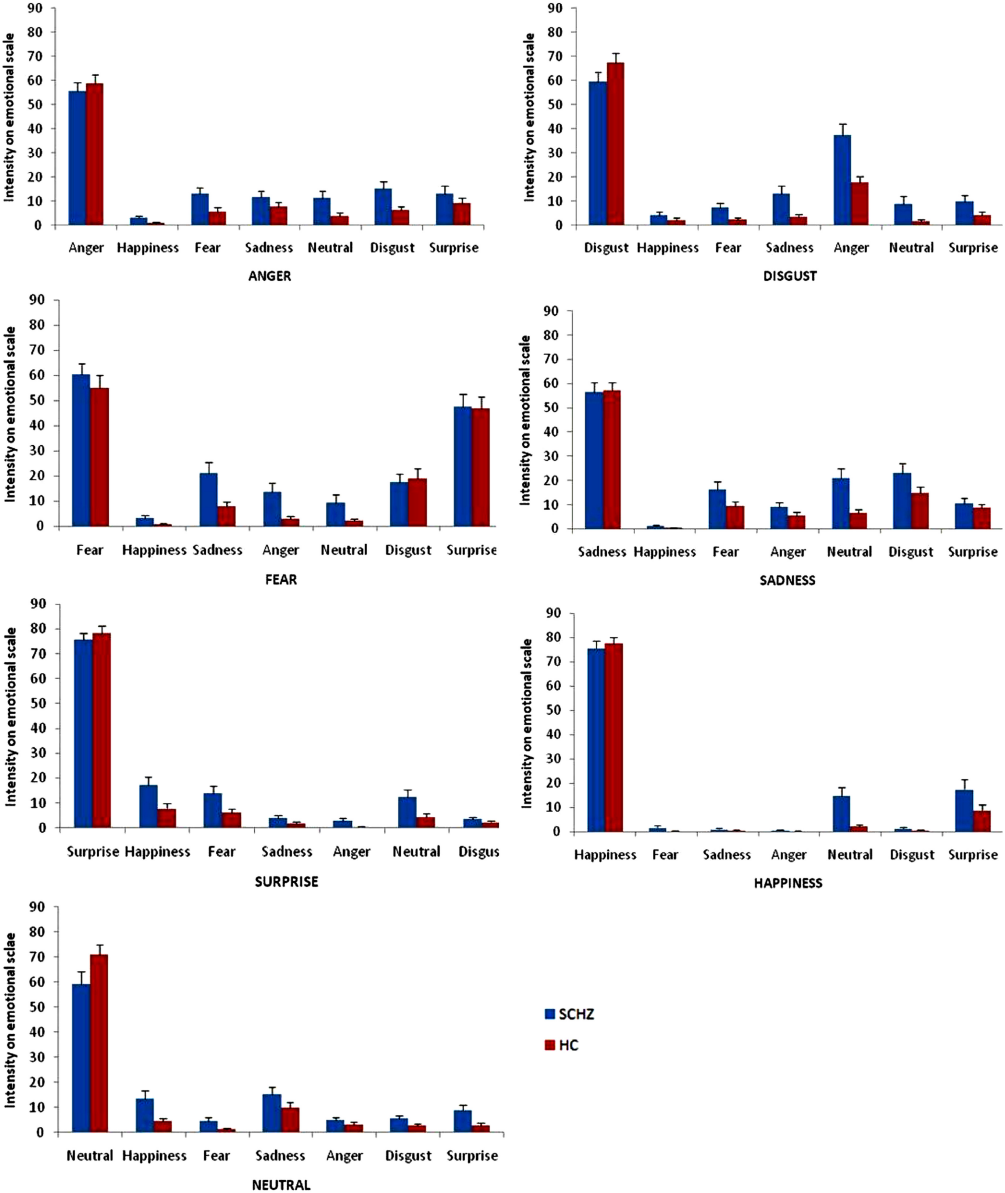
**Emotional biases on target and non-target scales for each category of emotional stimuli in the facial emotion recognition task.** Error bars indicate standard errors of mean.

***Facial recognition emotion task (Figure 3).*** The significant differences between the two groups on intensity ratings of target emotions on the non-target scales are shown in **Figure 3**. We found a Group × Emotion × Scale interaction, *F*(36,1584) = 4.21, *p* < 0.0001, partial eta-squared (η^2^) = 0.09. Analysis revealed main effects of emotion, *F*(6,264) = 23.97, *p* < 0.001, partial η^2^ = 0.35, group, *F*(1,44) = 6.50, *p* = 0.01, partial η^2^ = 0.13, and scale, *F*(6,264) = 18.26, *p* < 0.001, partial η^2^ = 0.29, and an Emotion × Scale interaction, *F*(36,1584) = 273.55, *p* < 0.001, partial η^2^ = 0.86. There was no Group × Emotion interaction, *F*(6,264) = 1.59, *p* = 0.15, partial η^2^ = 0.03, and no Group × Scale interaction, *F*(6,264) = 0.85, *p* = 0.53, partial η^2^ = 0.02. Results of the extended statistical analyses for each stimulus and each scale are reported next.* Anger stimulus*. Ratings on the Anger Scale showed no significant difference between SCHZ and HC [*F*(1,44) = 1.77, *p* = 0.19]. Contrasts showed that SCHZ rated the Neutral scale [*F*(1,44) = 5.41, *p* = 0.02], the Happiness Scale [*F*(1,44) = 7.63, *p* = 0.008], the Fear scale [*F*(1,44) = 6.41, *p* = 0.01] and the Disgust scale [*F*(1,44) = 8.34, *p* = 0.006] significantly higher than HC did. *Disgust stimulus*. Rating on the Disgust Scale showed no significant difference between SCHZ and HC, [*F*(1,44) = 1.90, *p* = 0.18]. Contrasts showed that SCHZ rated the Neutral scale [*F*(1,44) = 5.33, *p* = 0.03], the Fear scale [*F*(1,44) = 6.11, *p* = 0.02], the Sadness scale [*F*(1,44) = 9.19, *p* = 0.004] and the Anger scale [*F*(1,44) = 13.71, *p* < 0.001] significantly higher than HC did. *Fear stimulus*. Rating on the “Fear” scale showed no significant difference between SCHZ and HC, [*F*(1,44) = 0.65, *p* = 0.42]. Contrasts showed that SCHZ rated the Neutral scale [*F*(1,44) = 4.25, *p* = 0.045], Anger scale [*F*(1,44) = 8.99, *p* = 0.004], Happiness scale [*F*(1,44) = 5.07, *p* = 0.03] and the Sadness scale [*F*(1,38) = 8.92, *p* = 0.005] significantly higher than HC did. *Happiness stimulus*. Rating on “Happiness” scale, contrasts showed no significant difference between SCHZ and HC [*F*(1,44) = 0.25, *p* = 0.61]. Contrasts showed that SCHZ rated the Neutral scale [*F*(1,44) = 11.99, *p* = 0.001] significantly higher than HC did. *Neutral stimulus*. Rating on “Neutral” scale, contrasts showed no significant difference between SCHZ and HC [*F*(1,44) = 3.47, *p* = 0.07]. Contrasts showed that SCHZ rated the Happiness scale [*F*(1,44) = 7.36, *p* = 0.009], Fear scale [*F*(1,44) = 5.87, *p* = 0.02], Disgust scale [*F*(1,44) = 5.35, *p* = 0.03] and the Surprise Scale [*F* (1,44) = 5.24, *p* = 0.03] significantly higher than HC did. *Sadness stimulus*. Rating on the “Sadness” scale showed no significant difference between SCHZ and HC, [*F*(1,44) = 0.2, *p* = 0.90]. Contrasts showed that SCHZ rated the Neutral scale [*F*(1,44) = 3.35, *p* = 0.03] significantly higher than HC did. *Surprise stimulus*. Rating on “Surprise” scale showed no significant difference between SCHZ and HC [*F*(1,44) = 0.44, *p* = 0.51]. Contrasts showed that SCHZ rated the Neutral scale [*F*(1,38) = 5.24, *p* = 0.03], Happiness scale [*F*(1,44) = 6.13, *p* = 0.02] and the Fear Scale [*F*(1,44) = 5.55, *p* = 0.02] significantly higher than HC did.

**FIGURE 4:**
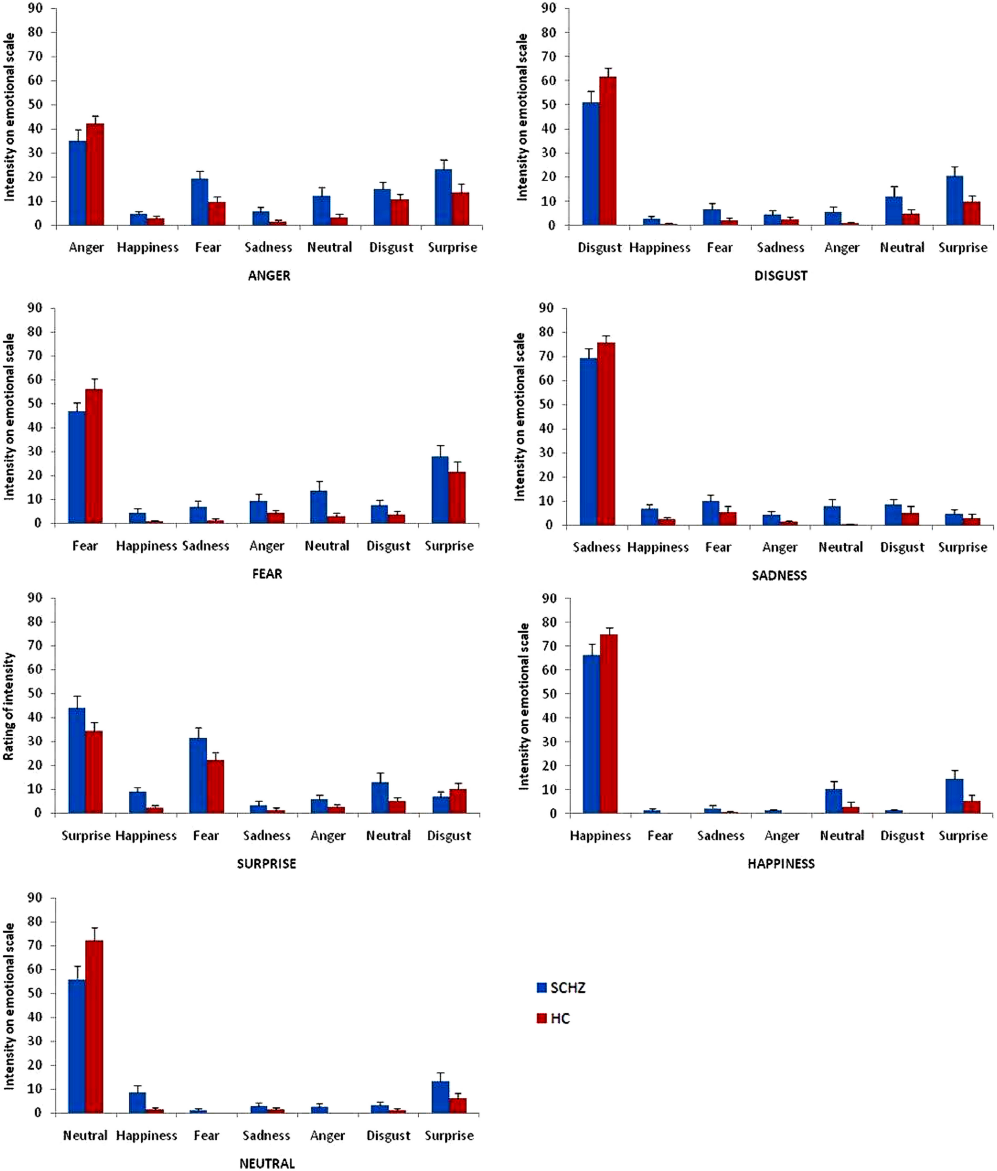
**Emotional biases on target and non-target scales for each category of emotional stimuli in the vocal emotion recognition task.** Error bars indicate standard errors of mean.

***Vocal recognition emotion task (Figure 4).* Figure 4** shows the significant differences between patients and HCs on intensity ratings of target emotions on the non-target scales. We found a Group × Emotion × Scale interaction, *F*(36,1584) = 3.68, *p* < 0.001, partial η^2^ = 0.08. Analyses revealed main effects of emotion, *F*(6,264) = 4.96, *p* < 0.001, partial η^2^ = 0.10, and scale, *F*(6,264) = 11.53, *p* < 0.001, partial η^2^ = 0.21, and Emotion × Scale, *F*(36,1584) = 198.95, *p* < 0.001, partial η^2^ = 0.82, Group × Emotion, *F*(6,264) = 3.28, *p* = 0.003, partial η^2^ = 0.07, and Group × Scale, *F*(6,264) = 2.25, *p* = 0.04, partial η^2^ = 0.05 interactions. There was no effect of group, *F*(1,44) = 3.02, *p* < 0.09, partial η^2^ = 0.06. Results of the extended statistical analyses for each stimulus and each scale are reported next. *Anger stimulus*. Rating on “Anger” scale showed no significant difference between SCHZ and HC [*F*(1,44) = 1.77, *p* = 0.19]. When the stimulus was “Anger,” contrasts showed that SCHZ rated the Neutral [*F*(1,44) = 5.37, *p* = 0.03], Fear scale [*F*(1,44) = 6.48, *p* = 0.01] and the Sadness scale [*F*(1,44) = 4.59, *p* = 0.04] significantly higher than HC did. *Disgust stimulus*. Rating on “Disgust” scale, contrasts showed no significant difference between SCHZ and HC, [*F*(1,44) = 3.34, *p* = 0.07]. Contrasts showed that SCHZ rated the Happiness [*F*(1,44) = 3.67, *p* = 0.06], Anger scale [*F*(1,44) = 5.01, *p* = 0.03] and the Surprise scale [*F*(1,44) = 4.52, *p* = 0.04] significantly higher than HC did. *Fear stimulus*. Rating on “Fear” scale, contrasts showed no significant difference between SCHZ and HC, [*F*(1,44) = 2.56, *p* = 0.12]. Contrasts showed that SCHZ rated the Neutral scale [*F*(1,44) = 6.63, *p* = 0.01], Happiness scale [*F*(1,44) = 5.43, *p* = 0.02] and the Sadness scale [*F*(1,44) = 5.48, *p* = 0.02] significantly higher than HC did. *Happiness stimulus*. Rating on “Happiness” scale showed no significant difference between SCHZ and HC [*F*(1,38) = 2.39, *p* = 0.13]. Contrasts showed that SCHZ rated the neutral scale [*F*(1,38) = 4.55, *p* = 0.04] and the Surprise scale [*F*(1,44) = 5.03, *p* = 0.03] significantly higher than HC did. *Neutral stimulus*. Rating on “Neutral” scale showed significant difference between SCHZ and HC [*F*(1,44) = 4.54, *p* = 0.04]. Contrasts showed that SCHZ rated the Happiness scale [*F*(1,38) = 6.38, p = 0.02] significantly higher than HC did. *Sadness stimulus*. Rating on “Sadness” scale showed no significant difference between SCHZ and HC, [*F*(1,44) = 1.59, *p* = 0.21]. Contrasts showed that SCHZ rated the Neutral scale [*F*(1,44) = 7.43, *p* = 0.009] and the Happiness Scale [*F*(1,44) = 4.31, *p* = 0.04] significantly higher than HC did. *Surprise stimulus*. Rating on “Surprise” scale showed no significant difference between SCHZ and HC [*F*(1,44) = 2.22, *p* = 0.14]. Contrasts showed that SCHZ rated the Happiness scale [*F*(1,44) = 9.69, *p* = 0.003] significantly higher than HC did.

### VERSION A VERSUS VERSION B COMPARISON

No significant difference was found between the accuracy (percentage of correct responses) in the HC group for version A and B for facial recognition task (*t* = 0.50;* p* = 0.62) and vocal recognition task (*t =*-0.23;* p* = 0.82).

### CORRELATIONS BETWEEN EMOTION RECOGNITION RESULTS AND CLINICAL VARIABLES IN THE PATIENTS WITH SCHIZOPHRENIA

We found a significant correlation between the PANSS scores and the overall scores on the facial (*r* = -0.42; *p* = 0.04) and vocal (*r* = -0.43; *p* = 0.04) emotion recognition tasks. More specifically, scores on the PANSS positive subscale were correlated with overall scores on the facial (*r* = -0.54; *p* = 0.008) and vocal (*r* = -0.60; *p* = 0.002) recognition tasks, and scores on the PANSS general psychopathology subscale were correlated with overall scores on the facial (*r* = -0.51; *p* = 0.01) and vocal (*r* = -0.55; *p* = 0.006) recognition tasks.

Spearman’s correlation coefficients showed a significant correlation between digit span forward and the overall score on the vocal emotion recognition task (*r* = 0.58; *p* = 0.004), and between perseverative errors on the MCST and the overall score on the facial emotion recognition task (*r* = 0.43; *p* = 0.04). None of the other cognitive tests were shown to be related to the overall scores.

## DISCUSSION

In this study, we sought to pinpoint the presence of emotional biases in both facial and vocal emotion recognition in chronic schizophrenia, controlling for confounding factors. In both the visual and vocal modalities, our main findings pointed to emotional biases in the patients’ ratings of emotional intensity of all the target emotions.

Using an original emotion recognition procedure that had already been validated (Péron et al., 2010, 2011), we compared a group of 23 patients with chronic schizophrenia and 23 HCs on the recognition of emotion in both visual and vocal modalities. We used two complementary methods to analyze performances on the two emotional tasks. In what was effectively a forced-choice procedure, participants produced the categorical judgments that are classically used in the literature. Thus, in our first analysis, we compared the percentages of correct responses provided by the two groups in order to assess these categorical judgments. Our second analysis allowed us to describe the emotion recognition biases in greater depth, by looking at the intensity ratings provided for each emotional category. Results for categorical judgments (i.e., number of correct responses expressed as a percentage of total responses) for the two emotional tasks revealed that patients with schizophrenia performed more poorly than HCs. However, we failed to demonstrate an effect of modality on emotional recognition impairments for patients with schizophrenia. Very few studies have compared the effect of modality (vocal vs. visual) on emotion recognition, and their designs are too heterogeneous to draw clear conclusions.

At a second level of analysis, the patients with schizophrenia were found to be less discriminative than HCs in their intensity ratings for both modalities (facial and vocal). Although they provided the same intensity ratings on the target scales (i.e., categorical judgments), they responded differently on the non-target scales thus exhibiting biases in their responses. These results are consistent with previous studies reporting emotional biases (Kohler et al., 2003) in chronic schizophrenia.

Delusions and hallucinations in schizophrenia may contribute to the introduction of biases. Patients with greater positive (including delusions and hallucinations) and general symptoms (including anxiety, attentional deficit, and depression) achieve lower overall recognition scores in the two emotional paradigms (vocal and facial). Kee et al. (1998) reported a relationship between positive symptoms and facial and vocal emotion identification, consistent with greater impairment of emotion recognition in paranoid patients (Pinkham et al., 2011).

Moreover, patients with poorer digit span forward scores had poorer overall scores on the facial emotion recognition task, while a higher number of perseverations on the MCST was related to a lower overall score on the vocal recognition task. Bozikas et al. (2004) reported an association between neuropsychological deficits (especially in executive functions) and facial and vocal emotion identification. Our results point to a cognitive difficulty in inhibiting non relevant information (introducing biases) that could explain the deficit in facial and vocal emotion recognition in schizophrenia.

The biases in emotion processing observed here can be explained in three ways. The first explanation refers to the sensory deficit in schizophrenia. Several studies have reported a basic sensory disturbance in the processing of emotion in the facial (Turetsky et al., 2007; Wynn et al., 2008; Norton et al., 2009; Laprevote et al., 2010; McBain et al., 2010; Bedwell et al., 2013; Jahshan et al., 2013) and vocal (Leitman et al., 2005, 2007, 2010, 2011a; Gold et al., 2012; Kantrowitz et al., 2013) modalities. This is unlikely to be the case in our study since patients with basic sensory disturbances, as assessed with the Benton test and the PEGA, were excluded from our sample. However, one cannot rule out that biases in our sample were the due to a deficit in multisensory integration.

The second explanation concerns the lack of cognitive control in schizophrenia (Chambon et al., 2008; Lesh et al., 2011; Kring and Elis, 2013). *Cognitive control* is defined as the ability to mobilize cognitive resources to process relevant information and inhibit irrelevant information in daily life situations. Anticevic and Corlett (2012) discussed interference between relevant and irrelevant emotional stimuli resulting in over-responsiveness to neutral stimuli. Similarly, in our study, we found that patients with schizophrenia provided extremely high intensity ratings on the neutral scale when they listened to neutral stimuli. In a recent study featuring an emotion identification task with gradual exposure to stimuli, Lee et al. (2011) found that patients with schizophrenia explored happy and fearful faces differently. In an fMRI study, Fakra et al. (2008) examined the brain activity of patients with schizophrenia while they performed emotional face matching and identification tasks. Comparisons between patients and HCs revealed similar patterns of brain activity for the identification task, whereas the matching task was characterized by a lack of amygdala activity in the patient group, with patients undertaking more cognitive exploration than the HCs. According to Fakra et al. (2008), focusing on specific facial features requires more cognitive resources than overall exploration, and could explain the lack of amygdala activity. Using implicit and explicit tasks, Roux et al. (2010) demonstrated impairment for the explicit recognition of emotional prosody, whereas the implicit processing of emotional prosody (emotional version of the Stroop Test) was preserved. This study highlighted the impact of cognitive control on the processing of emotional prosody. This is likely to be the case here, as we found a relationship between overall performances and an executive function deficit in our sample of patients with chronic schizophrenia. Others studies yielded some explanations as to the mechanism behind the emotional deficit in schizophrenia, and highlighted the impact of misusing cognitive resources in emotion processing (Bach et al., 2009; Christensen et al., 2013).

Third and last, the mutual influence of sensory disorder and cognitive control impairment in schizophrenia may explain the emotional disorders described in the literature. In a recent study using event-related potentials, Pinheiro et al. (2013) reported impairment in both the early sensory and late cognitive stages of emotional prosody processing in patients with chronic schizophrenia.

Schizophrenia is related to brain dysfunction, especially in the prefrontal striatonigral circuit. Some studies have shown that a dysfunction of the prefrontal cortex is related to the identification of emotional context in schizophrenia (Gur et al., 2007; Fakra et al., 2008; Leitman et al., 2011b). Using an emotional go/no go task, Vercammen et al. (2012) compared the fMRI brain activity of schizophrenia patients with that of healthy participants. Inhibiting responses to negative emotional stimuli elicited increased activity in a brain network including the prefrontal, cingulate and parietal cortices in healthy participants, but not in patients with schizophrenia. During the inhibition of responses to positive emotional stimuli, patients with schizophrenia exhibited greater activity in the dorsolateral prefrontal cortex than HCs. Other studies have reported a deficit in the balance of prefrontal cortex–amygdala activity in an easy emotion processing task (no cognitive load; Anticevic and Corlett, 2012) and an abnormal modulation in prefrontal-subcortical connectivity during a working memory task (Anticevic et al., 2012). Some studies have highlighted the role of the primary sensory cortex in the impairment of emotion recognition. Rolls et al. (2008) described a dynamic neural network in the frontal cortex with a stable state involved in several types of cognitive and emotion processing. This neural network can be affected by neural noise (i.e., stochastic neural firing), leading to maladaptive behavior that includes executive and emotional disorders. Moreover, the emotional noise observed in our study can be related to an imbalance between the prefrontal cortex and the basal ganglia. Péron et al. (2013), based on Graybiel (1997) work, developed a model wherein the basal ganglia are involved in the generation of patterns of brain activation related to habits (or engrams), of over-learned cognitive, motor, and emotional sequences in the context of emotional processing. These habits or engrams can be uploaded by the basal ganglia in order to adapt more quickly and more accurately. Graybiel (1997) postulated that the basal ganglia system plays a role in the generation of positive and negative symptoms in schizophrenia.

This study had several limitations that need to be addressed. First, only 23 patients were assessed. However, we took care to recruit patients with the same clinical characteristics, in order to construct a homogenous group. Second, as described by Salgado-Pineda et al. (2005), dopamine influences emotion recognition. Our results may thus have been skewed by the patients’ medication (antipsychotics and, for some of them, antidepressants). Furthermore, we did not control for the physical properties (pitch, intensity and timbre) of the vocal stimuli we used, even though it is these variations in physical properties that create the profiles of emotional prosodies (Banse and Scherer, 1996; Grandjean et al., 2006). Another limit might be that the emotional task order was not counterbalanced and the presentation of stimuli within a task was not randomized raising the issue of the practice effect of our tasks. However, all participants were familiarized with the procedure before the two emotional tasks. Practice effect had a major impact on reaction time which was not the main variable in the present study (Poulton, 1982). Moreover, others studies using similar tasks did not report practice effect (e.g., Bach et al., 2009; Fiszdon and Bell, 2009).

Further research is needed to investigate the involvement of cognitive control in the management of top-down and bottom-up processing, using both implicit and explicit emotion processing tasks.

In summary, this study showed that chronic schizophrenia induces emotional biases for all emotions in two sensory modalities (visual and vocal), and appears to cause interference in emotion recognition. There are now at least two mechanisms that need to be, considered if we are to explain impairments in emotion recognition: a deficit in sensory functions and a lack of cognitive control. These results could help to enhance current cognitive and emotional remediation in schizophrenia.

## Conflict of Interest Statement

The authors declare that the research was conducted in the absence of any commercial or financial relationships that could be construed as a potential conflict of interest.
